# Long-term efficacy and quality of life after antireflux surgery

**DOI:** 10.1007/s00464-025-11608-5

**Published:** 2025-02-18

**Authors:** Philip K. Bang, Naja H. Andersen, Frederik Hvid-Jensen, Niels Christian Bjerregaard, Daniel W. Kjaer

**Affiliations:** 1 Department of Anesthesiology and Intensive Care, Regional Hospital Viborg, Viborg, Denmark; 2 Department of Clinical Medicine, Aarhus University, Aarhus, Denmark; 3 Department of Surgery, Aarhus University Hospital, Aarhus, Denmark

**Keywords:** Gastro-esophageal reflux disease, GERD, Laparoscopic antireflux surgery, Fundoplication, Quality of life, Proton pump inhibitor

## Abstract

**Background:**

Antireflux surgery (ARS) has been found to be an effective treatment of gastro-esophageal reflux disease (GERD); however, the long-term effects are uncertain. This study aimed to evaluate the long-term efficacy of ARS on quality of life, symptom severity, and use of proton pump inhibitors (PPIs).

**Methods:**

A validated GERD Health-Related Quality of Life (GERD-HRQL) Questionnaire was sent to 419 patients who underwent ARS at Aarhus University Hospital from January 2012 to April 2020. Patient records were reviewed retrospectively. The Danish National Prescription Registry was used to collect data on the use of PPIs before and after ARS.

**Results:**

A response rate of 71% resulted in a total of 164 patients included in the study with a median follow-up time of 4.8 years (interquartile range: 2.5–6.7). The total GERD-HRQL median score at follow-up was 11.5 (IQR: 4–22). The proportion of patients experiencing daily symptoms of heartburn and regurgitation was significantly reduced pre- to postoperatively from 90 to 70% to 32% and 29%, respectively. Five years after surgery, 47% of patients had completely ceased PPI usage, while 44% were long-term users.

**Conclusion:**

A lasting long-term effect of ARS on GERD symptoms was found, although almost a third of patients still experience heartburn and/or regurgitation daily. Almost half of patients were not taking PPIs 5 years after ARS, but 44% became long-term users. Patients should be made aware that long-term PPI therapy often is necessary following ARS.

**Graphical Abstract:**

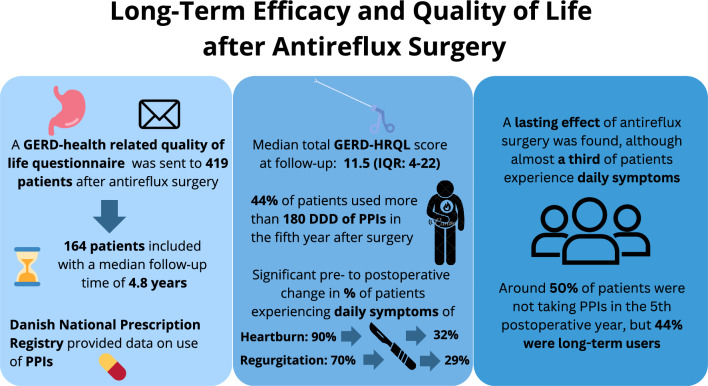

Gastro-esophageal reflux disease (GERD) is a condition which develops when reflux of stomach content into the esophagus causes symptoms, most commonly of heartburn and regurgitation, and/or complications [1]. Complications of GERD include reflux esophagitis, reflux stricture, and Barrett’s esophagus (BE), with the latter being the most important risk factor for adenocarcinoma of the esophagus [1, 2].

GERD is a highly prevalent condition worldwide with estimated prevalences of 8.8%−25.9% in Europe and 18.1%−27.8% in North America [3]. An estimated 22% of the 40–65-year-old Danish population report at least mild gastro-esophageal reflux symptoms [4]. GERD has been shown to significantly reduce quality of life (QoL) as well as productivity at work and results in increased absenteeism [4, 5].

Medical treatment of GERD aims to decrease the acidity of the stomach and consists primarily of proton pump inhibitors (PPIs) [6]. PPIs are generally well-tolerated and an effective treatment with healing rates of up to 94% on erosive esophagitis (EE) caused by GERD as well as complete resolution of heartburn in more than 60% of patients with EE [7]. Antireflux surgery (ARS) in the form of a laparoscopic fundoplication (LF) is a well-established alternative treatment option of GERD. Careful selection of patients to undergo surgery is essential, as the outcome of surgery depends on the correct identification of the disease. The strongest predictors of a good outcome of LF are an abnormal 24-h esophageal pH score, a typical primary symptom of GERD and clinical response to PPI therapy [8]. Therefore, all patients are prior to surgery evaluated with gastroscopy, impedance pH monitoring, and high resolution (HR) esophageal manometry [9]. Surgery is indicated when patients proven to need long-term treatment of GERD experience persistent troublesome symptoms, reduced QoL and/or progression of disease despite adequate PPI therapy [9].

A systematic Cochrane review of treatment of GERD found significantly better effect on heartburn, regurgitation, and GERD-specific QoL for patients treated with LF compared to medical management in the short and medium terms, while the proportion of dysphagia was higher in the LF group [10]. However, there is little evidence of the long-term effects on the GERD-related QoL of LF, with the review finding no significant difference beyond the short term compared to medical therapy [10]. Another recent systematic review similarly found superior short-term but not long-term QoL of patients treated surgically relative to patients treated medically [11]. This review did find a better long-term symptom control in surgically treated patients, although there was significant study heterogeneity and the only high-quality RCT included did not find improved long-term symptom control.

The reported use of PPIs after ARS varies between 15 and 51% with follow-up periods from 4 to 20 years [12–19]. A rate of PPI use of 28% is found in the systematic review [11]. The use of PPIs in most studies is not accounted for in details, however, a Danish population-based register study found a 5-year risk of becoming a long-term PPI user of 29% increasing to 57% at 15 years [20].

The present study aims to evaluate the long-term efficacy, patient-reported QoL, and use of PPIs in patients who underwent ARS in a single-center cohort.

## Methods

This study was a retrospective cohort study complying with the STROBE reporting guidelines. Electronic patient records (EPRs) as well as a follow-up questionnaire were utilized in assessing the long-term efficacy of ARS on GERD-related QoL and symptoms. The Danish National Prescription Registry (DNPR), which contains data on all prescription drugs redeemed by Danish citizens categorized according to the Anatomical Therapeutic Chemical (ATC) index, was used to collect data on PPI usage after ARS.

Questionnaires were sent and resent and data collected through October 2020 to June 2021. Approval for the study was obtained from the local Hospital Administration and the Central Region of Denmark. Patients provided informed consent to participate in the study by responding to the questionnaire.

### Study population

All adult patients (age ≥ 18) who underwent ARS for GERD at the Department of Surgery at Aarhus University Hospital (AUH) from 1st January 2012 to 30th April 2020 were identified by searching the procedure code “Laparoscopic gastro-esophageal reflux surgery” (KJBC01) in the hospital’s electronic registry (Business Intelligence Portal). The procedure code includes redo-antireflux surgery as well as surgeries, where a fundoplication was performed as part of a surgery with the primary indication being large, symptomatic hiatal hernias. All patients were sent an online QoL questionnaire via e-Boks, which is an online digital mailbox linked to the Central Person Registry (CPR) number, a unique identifier assigned to all Danish citizens. All non-responders were sent another questionnaire after approximately three months. Patients exempt from using e-Boks received a letter.

Excluded from the study were all patients who had previously undergone antireflux surgery, as well as patients who had endoscopically or computed tomography verified large hiatal hernia as well as either not having undergone pH monitoring or having a normal reflux index at pH monitoring prior to surgery, as these patients were operated primarily due to a symptomatic large hiatal hernia without GERD, where the surgery included an additional fundoplication.

### Data collection

Baseline patient characteristics, operative and postoperative characteristics were retrieved from retrospective review of the EPRs. Index date was the date of surgery and the follow-up period was until response to the questionnaire.

Data on all redeemed prescriptions were extracted from the DNPR from one year prior to the surgery until November 2023. The use of PPIs (ATC: A02BC) was quantified as the amount of defined daily doses (DDD) redeemed after surgery. The DDD use per year from one year before the date of surgery until November 2023 was calculated. Deceased patients contributed with data from date of surgery to date of death, if the date of death was at least one year after surgery.

The presence of a hiatal hernia, reflux esophagitis, and BE was recorded at gastroscopy. Motility was characterized as either normal or as ineffective, if there was evidence of a non-specific esophageal motility disorder on HR manometry. Patients discontinued PPI intake 5 days prior to impedance pH monitoring. Pathological acid reflux was defined as a reflux index of > 5%. Volume reflux was defined as > 73 total reflux episodes with positive symptom correlation.

As part of preoperative evaluations, all patients filled out a questionnaire regarding the duration of symptoms, grading their GERD symptoms on a scale of 0–3, where 0 equals no symptoms and 3 indicates daily symptoms, as well as an overall severity grading of their symptoms on a scale of 1–10 with 10 being the most severe symptoms.

The type of fundoplication performed was left to the surgeon’s preference and followed standard surgical technique.

Postoperative objective assessments by gastroscopy, HR manometry, and pH monitoring were not performed routinely, but only as indicated if patients were experiencing symptoms and were considered for reoperation.

**Fig. 1:**
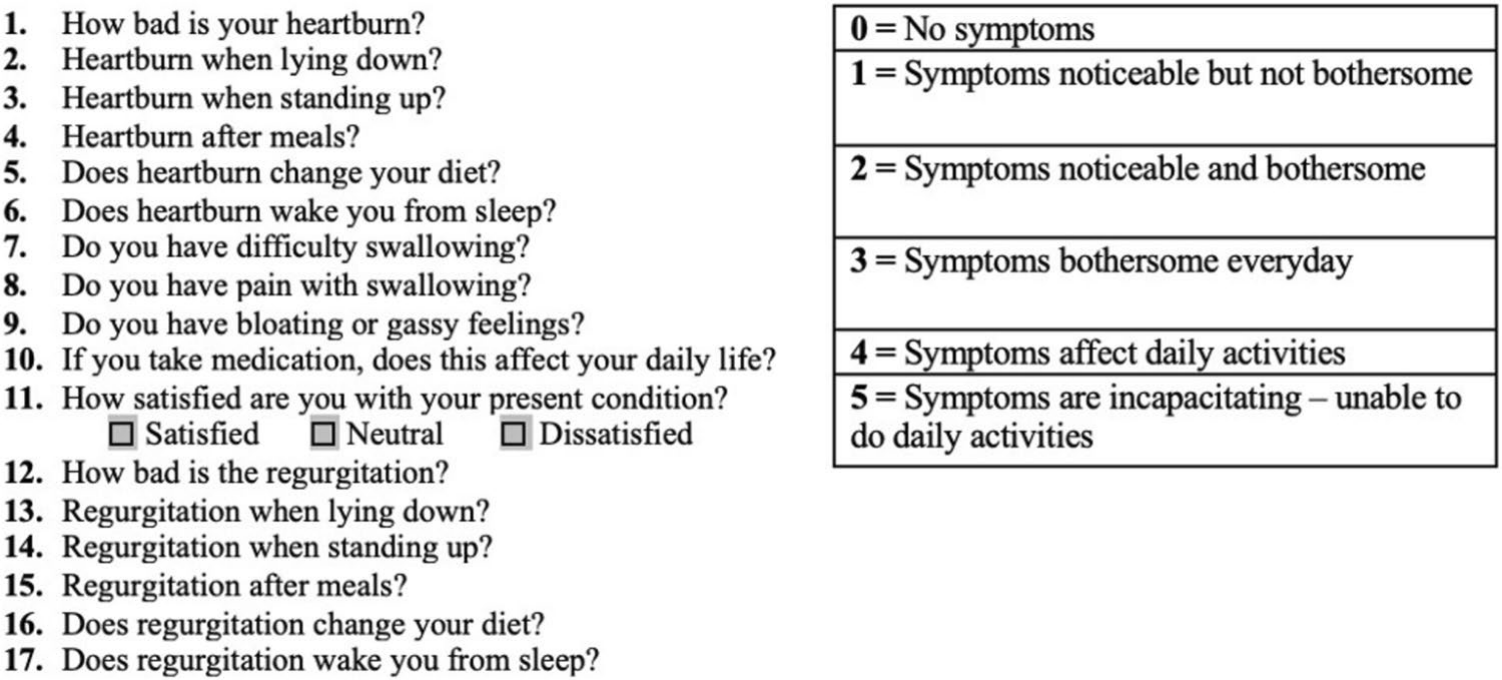
Modified GERD-HRQL questionnaire (questions 1–10 contribute to the total GERD-HRQL score, questions 11–18 are supplemental and not included in the total score)

At follow-up, all patients were sent a validated GERD Health-Related Quality of Life Questionnaire (GERD-HRQL) [21]. This is a 10-item instrument, where patients grade their GERD-related symptoms from 0 (no symptoms) to 5 (incapacitating symptoms), resulting in a total score of maximally 50 points. Patients were additionally asked to similarly grade their regurgitation symptoms and state whether they were satisfied, neutral, or dissatisfied with their current condition (Fig. 1).

### Statistical analysis

Variables were summarized using descriptive statistics. Continuous variables were tested for normal distribution using Q–Q plots and were presented as a mean with standard deviation if the variable was normally distributed and as a median accompanied by the interquartile range (IQR) if it were not. McNemar’s test was used for testing statistical significance in paired nominal data. A *p* value of < 0.05 was considered statistically significant.

Statistical analyses were performed using Stata 16.1.

## Results

### Study population

**Fig. 2:**
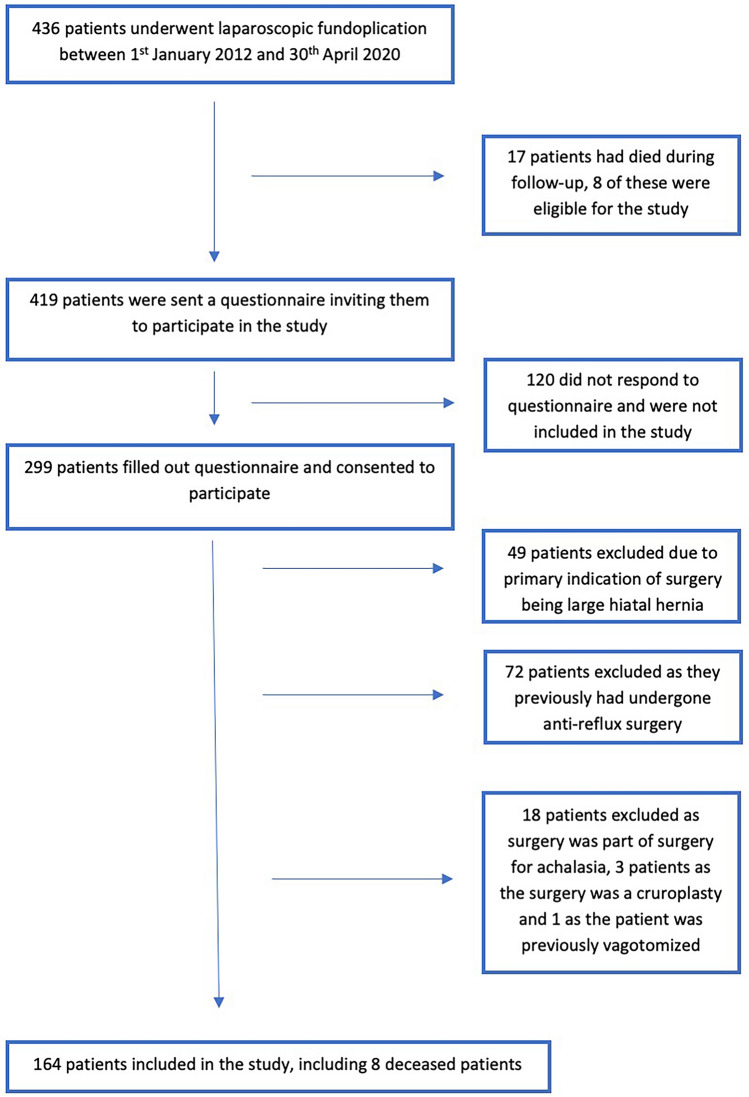
Flowchart of study population

Between 1st January 2012 and 30th April 2020, 436 patients who underwent ARS at AUH were identified. Of these, 17 patients were deceased, eight of which were eligible for the study. One patient died 3 days after surgery. The patient was discharged one day after an uneventful postoperative course, and the cause of death was not established. A total of 419 patients were sent a questionnaire of which 299 (71%) replied. Excluded were 72 patients (23%) due to previously having undergone ARS, 49 (16%) as the primary indication for surgery was a large hiatal hernia, and 22 patients (7%) were excluded as the surgery performed was either part of a myotomy for achalasia (18 patients), consisted only of a cruroplasty procedure (three patients) or due to previously being vagotomized (one patient). In total, 164 patients were included in the study with 156 respondents to the questionnaire (Fig. 2).

The median follow-up time was 4.8 years (IQR: 2.5–6.7 years, range: 0.4–8.7 years). The baseline characteristics of the study population are presented in Table 1, and the operative and postoperative characteristics are presented in Table 2.

**Table 1:** Baseline patient characteristics

Characteristic	*n* = 164
Mean age, years (SD)	51.5 (13.1)
Sex	
Male	77 (47%)
Female	87 (53%)
Mean body mass index (BMI) (kg/m^2^), (SD)	28.52 (4,5)
BMI 18.5–24.99	38 (23%)
BMI 25–29.9	71 (43%)
BMI > 30	55 (34%)
Smoking status	*n* = 162
Non-smoker	74 (45%)
Smoker	36 (22%)
Ex-smoker	52 (32%)
Alcohol consumption	*n* = 162
< 7/14 Units^a^ weekly	153 (94%)
> 7/14 Units^a^ weekly	9 (6%)
CCI, Total score	
0	60 (37%)
1	42 (26%)
2	40 (24%)
3	11 (7%)
4	8 (5%)
5	2 (1%)
> 5	1 (1%)
Duration of symptoms	*n* = 125
< 12 Months	5 (4%)
1–3 Years	34 (27%)
> 3 Years	86 (69%)
Preoperative gastroscopy	
Normal	22 (13%)
Hiatus hernia	125 (76%)
Reflux esophagitis	65 (40%)
Barrett’s esophagus	26 (16%)
Not performed	3 (2%)
Preoperative manometry	
Normal	96 (59%)
Ineffective motility	63 (38%)
Not performed	5 (3%)
Preoperative PH monitoring	
Normal	6 (4%)
Acid reflux	142 (87%)
Volume reflux	13 (8%)
Not performed	7 (4%)
Median reflux index, % (IQR)	10.4 (7.9–16.1)

*SD* Standard deviation, *A* one unit of alcohol defined as 12 g of alcohol. 7 units limit for women, 14 units limit for men as per recommendations from the Danish Health authorities, *CCI* Charlson comorbidity index, *IQR* Interquartile range

**Table 2:** Operative and postoperative characteristics

Characteristic	*n* = 164
Type of operation	
Nissen	111 (68%)
Toupet	53 (32%)
Conversion to open surgery	
YES	1 (0.6%)
NO	163 (99%)
Duration of surgery, minutes, median (IQR)	64 (51–87)
Length of hospital stay, days, median (IQR)	1 (1–1)
Preoperative complications	
Capnothorax	7 (4%)
Perforation of ventricle	0
Perforation of esophagus	0
Splenic bleeding	2 (1%)
Non-splenic bleeding	20 (12%)
None	139 (85%)
Clavien–Dindo classification	
GRADE 0	141 (86%)
GRADE I	13 (8%)
GRADE II	4 (2%)
GRADE IIIA	3 (2%)
GRADE IIIB	3 (2%)
30-day complications	
Perforation	2 (1%)
Bleeding	0%
Infection	4 (2%)
Death	1 (0.6%)
None	157 (96%)
Reoperation	
Yes	15 (9%)
No	149 (91%)
Postoperative gastroscopy during follow-up	
YES	83 (51%)
NO	81 (49%)
Postoperative gastroscopic dilatation	
Yes	30 (18%)
No	134 (82%)
Median days since surgery until first dilatation (IQR)	103 (61–292)
Median number of total dilatations if dilated (IQR)	2 (1–3)

*IQR* Interquartile range

### Symptoms and quality of life

**Fig. 3:**
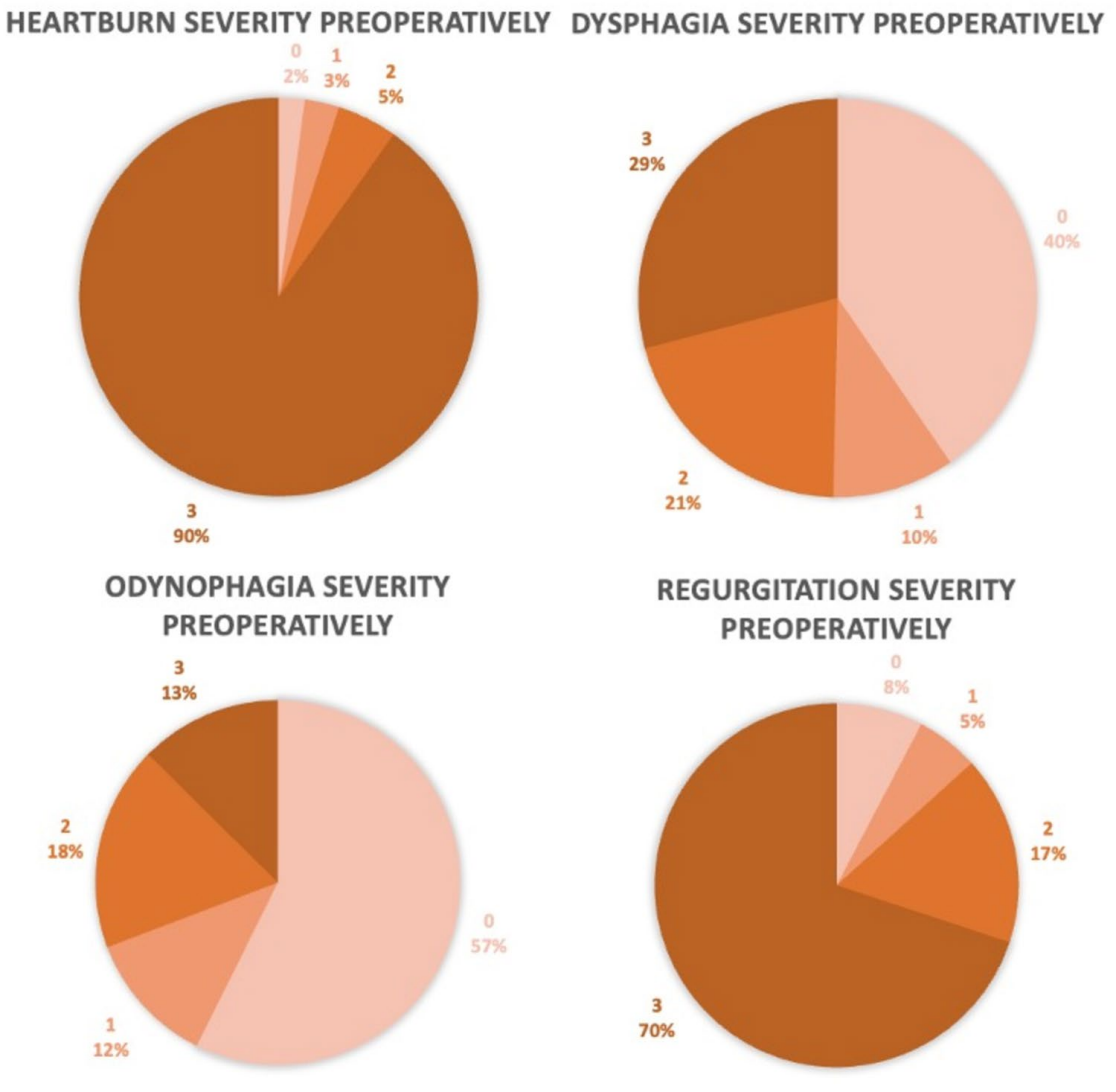
Preoperative symptom severity ratings on a scale of 0–3

**Fig. 4:**
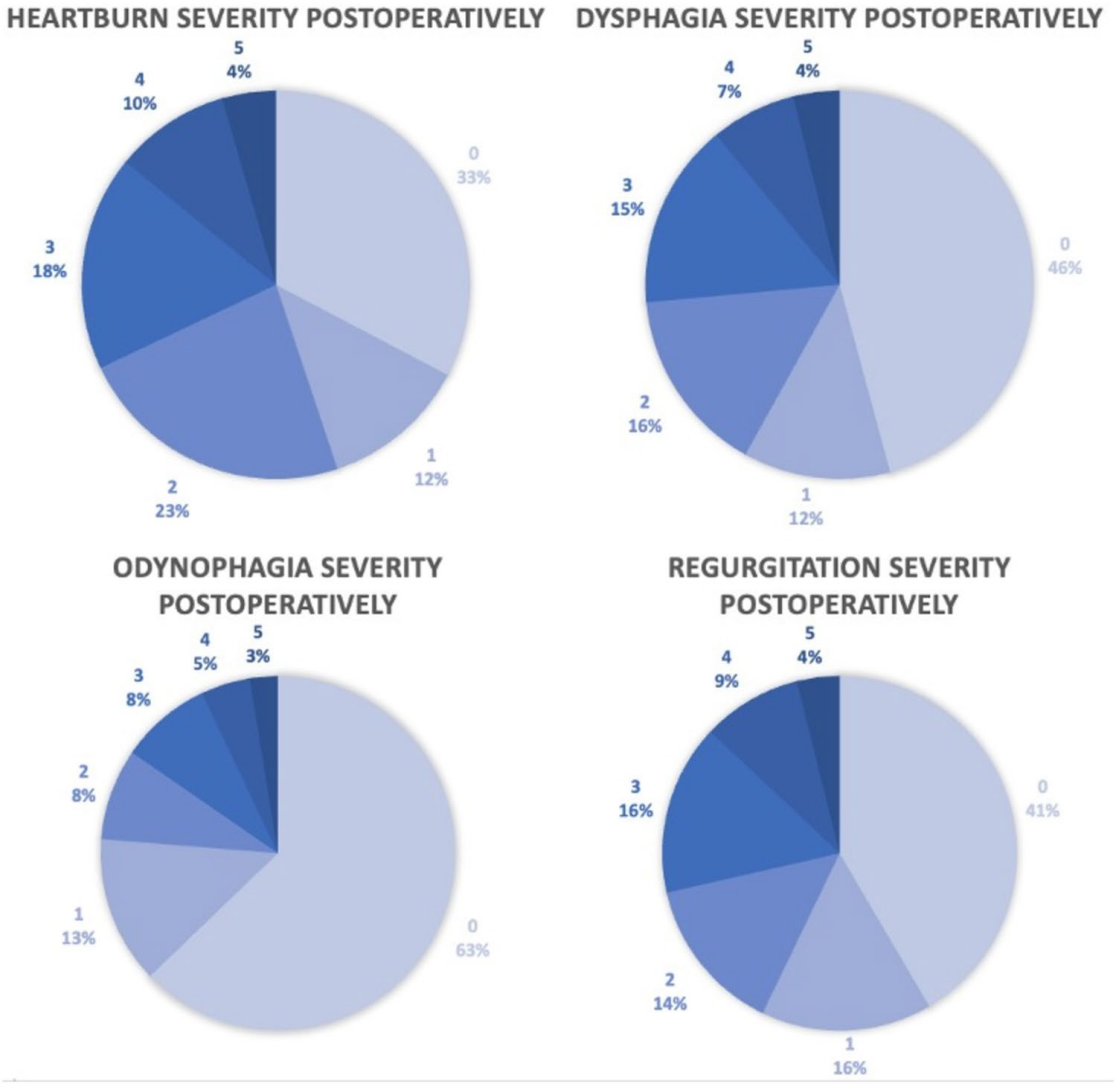
Postoperative symptom severity as rated on GERD-HRQL questionnaire questions 1, 7, 8, and 12

The preoperative severity of symptom ratings on a scale of 0–3 are presented in Fig. 3, and the postoperative symptom severity as rated on the GERD-HRQL scale of 0–5 in Fig. 4. Fifteen patients had missing preoperative symptom evaluations. These patients were not included in the paired data analysis. Four patients had missing values on up to four questions on the GERD-HRQL questionnaire for a total of nine missing values. These were handled by single mean imputations.

The median preoperative overall patient-rated severity of symptoms was 8 (IQR: 8–10). The total GERD-HRQL median score at follow-up was 11.5 (IQR: 4–22).

The median rating of heartburn severity preoperatively on a scale of 0–3 was 3 (IQR: 3–3). Regurgitation median severity was also rated 3 (IQR: 2–3) preoperatively, while dysphagia and odynophagia were rated 1 (IQR: 0–3) and 0 (IQR: 0–2), respectively. Postoperatively, on the GERD-HRQL questionnaire on a scale of 0–5, the median severity of heartburn rating was 2 (IQR: 0–3), regurgitation was rated 1 (IQR: 0–3), while dysphagia and odynophagia had a median rating of 1 (IQR: 0–3) and 0 (IQR: 0–1), respectively.

The proportions of patients experiencing daily symptoms, as indicated by a rating of 3 preoperatively or 3 or above postoperatively on the GERD-HRQL questionnaire are presented in Table 3. The proportion of patients experiencing daily heartburn was significantly reduced from 90% preoperatively to 32% postoperatively. For regurgitation, the proportion was also significantly reduced from 70% preoperatively to 29% postoperatively. Twenty-nine percent of patients were experiencing daily symptoms of dysphagia while 26% of patients did so postoperatively. Odynophagia was present daily in 13% preoperatively and 16% postoperatively. The pre- to postoperative changes in dysphagia and odynophagia were not statistically significant.

**Table 3:** Percentage of patients experiencing daily symptoms pre- and postoperatively. Mcnemar’s test used for testing statistically significant change

Symptom	Preoperative	Postoperative (N = 156)	*P*
Heartburn	90% (*n* = 143)	32%	< 0.0001
Dysphagia	29% (*n* = 143)	26%	0.7855
Odynophagia	13% (*n* = 141)	16%	0.1944
Regurgitation	70% (*n* = 143)	29%	< 0.0001

Fifty percent of patients rated their satisfaction with their current condition at follow-up as satisfied, 25% as neutral and 25% as dissatisfied. Seventy percent of patients would choose to undergo surgery again, 26% were not sure, while 4% would choose not to undergo surgery again.

### Use of PPIs

In the 5th year after surgery, 44% of patients had redeemed prescriptions on PPIs corresponding to a DDD of > 179 and as such were considered long-term users of PPIs (Table 4). Forty-seven percent of patients had not redeemed prescriptions on PPIs during the 5th postoperative year. In the year prior to surgery, 84% redeemed prescriptions on > 179 DDD, while 6% did not collect any PPI prescriptions.

**Table 4:** Yearly pre- and postoperative use of PPIs in percentage of patients in intervals of DDD

	One year preoperatively	Postoperative year				
DDD		0–1 (*n* = 162)	1–2 (*n* = 162)	2–3 (*n* = 162)	3–4 (*n* = 147)	4–5 (*n* = 133)
0	6%	43%	53%	48%	51%	47%
1–89	4%	8%	7%	4%	3%	4%
90–179	7%	14%	6%	10%	5%	5%
> 179	84%	36%	33%	38%	41%	44%

*PPI* Proton pump inhibitors; *DDD:* defined daily doses

## Discussion

This study found a median total GERD-HRQL score of 11.5 (IQR: 4–22) after a median of 4.8 years of follow-up. A statistically significant reduction in the proportion of patients experiencing daily symptoms of heartburn and regurgitation was found. At follow-up, 32% of patients were experiencing heartburn daily, 29% had daily symptoms of regurgitation, and 26% of dysphagia. In the 5th postoperative year, 44% of patients were taking 180 DDD or more of PPIs and as such were termed long-term PPI users. The reoperation rate was 9% and 50% of patients were satisfied with their current condition at follow-up, 25% were unsatisfied, while 25% were neutral. Seventy percent would choose surgery again, 4% would not and 26% were unsure.

As highlighted in the systematic review by McKinley et al. [11], evaluation of the surgical treatment of GERD is characterized by large heterogeneity and the use of different assessments of endpoints, which can make reliable conclusions difficult. This study sought to contribute with long-term data on well-defined endpoints using a validated questionnaire in the evaluation of symptoms and QoL as well as an accurate estimation of the use of PPIs.

The GERD-HRQL score of 11.5 found in this study is higher than previously reported in the literature. A similar retrospective study with a mean follow-up time of 8 years found a median GERD-HRQL score of 6.5 after ARS [14], while another similarly designed study found a mean GERD-HRQL rating of 5.7 at 5 years of follow-up, although this study utilized a different, 9-item version of the GERD-HRQL questionnaire resulting in a maximal score of 45 points [22]. Nikolic et al. [19] found, in a highly specialized setting, a median score of 2 after 4 years of follow-up. In the present study, although symptoms of heartburn and regurgitation significantly decreased, almost a third of patients experienced daily symptoms. These rates are higher than previously reported, with findings of daily symptoms of heartburn in 11% and regurgitation in 20% at 8.8-year follow-up in a retrospective study by Prassas et al. [23], while Dallemagne et al. [24] found that 90% of patients were free from recurrence of heartburn and reflux symptoms at 10-year follow-up. The rate of daily dysphagia at 26% is slightly lower than that of 32% found by Prassas et al. [23], but markedly higher than the 11% found in a randomized controlled trial (RCT) by Galmiche et al. [25] and the 2% found by Nikolic et al. [19].

The patients’ satisfaction with their current condition after surgery is somewhat lower than found in the literature, where between 71 and 90% report to be satisfied [13–15, 17, 19, 22, 24]. Seventy percent of patients would choose to undergo surgery again, a rate slightly lower than rates of 79%−88% previously found [19, 22].

This study found that 44% of patients had taken up long-term use of PPIs 5 years after surgery, while 47% did not take any PPIs. The use of a validated nationwide register containing data on all redeemed prescriptions is a major strength of this study, provides a precise estimate of PPI use and circumvents recollection bias. PPIs are available as an over-the-counter drug in Denmark, but only in non-reimbursed, low-dose small packages and 98% of PPI sale in Denmark is related to prescriptions [20].The reported rates of long-term PPI use after ARS are varied in the literature. In highly specialized settings, daily PPI use ranges from 13 to 17% [16, 19], while results from clinical trials range between 15 and 44% in the REFLUX and LOTUS trial [25, 26]. A recent systematic review found 28% of patients used PPIs at follow-up [11]. The clinical trials most often report the use of PPIs at monthly or annual visits and it is possible that use of PPIs in between visits were not accounted for. Being part of a clinical trial where the use of PPIs is considered a failure also introduces potential biases, as patients may be less likely to report or to take up PPI use. In other cohort studies, higher rates of PPI use are reported ranging from 15 to 51% [12, 13, 17]. A Danish nationwide, register-based study of PPI use after ARS found 5-, 10-, and 15-year risks of long-term PPI use of 29%, 41% and 57%, respectively, with the 5-year risk doubling from 22% at the beginning of the study period (1996–2000) to 43% at the end (2006–2010), a risk equivalent to the 5-year risk found in the present study. Interestingly, a similar Swedish nationwide, register-based study found only 15% of patients had taken up long-term PPI use at more than 5 years of follow-up [27].

The long-term use of PPIs after ARS is not necessarily equal to failure of treatment and a poor correlation between PPI use and objective findings and symptoms has previously been established [28].

ARS may still have benefitted patients in spite of them taking up long-term PPI use, if the combination of surgery and PPIs resulted in adequate relief of symptoms that preoperatively were incapacitating. The vast majority of patients taking up long-term PPI use can most likely be attributed to some degree of the same reflux symptoms that indicated ARS to begin with. Other reasons may be present for the reuptake of PPIs use such as prophylaxis in relation to glucocorticoid treatment, bloating, and stomach ulcers [15], while others may take them in spite of experiencing no symptoms when not taking PPIs [17].

A reoperation rate of 9% was found, similar to a Danish nationwide register-based study, which found 5- and 10-year reoperation rates of 9.3% and 11.7% [29]. These rates are somewhat higher than rates of 3% after more than 5 years in a nationwide Swedish study [27], 5% after 5 years in a large study from the USA [30], but similar to rates of 11% found by Pascotto et al. [14]. Interestingly, a Danish nationwide study reported a reoperation rate of 5% between 1997 and 2005 [31]. As the reoperation rate in Denmark have almost doubled since, it may reflect a less conservative selection of patients for reoperation, which might also contribute to the higher reoperation rate found in the present study compared to international studies.

A low postoperative complication rate, a short hospital stay and a 30-day all-cause mortality rate of 0.6% was found, similar to that of nationwide studies [27, 29, 31] proving ARS as a safe treatment.

This study is limited by its retrospective design, relying on patients responding to the GERD-HRQL questionnaire in order to be included in the study. A response rate of 71% was obtained and is markedly higher than that of similar studies, where response rates of 49%–54% were found [22, 23], although lower than the near complete follow-up of 96% by Pascotto et al. [14]. The fact that data are collected retrospectively from EPRs is another limitation, resulting in some missing data. On the preoperative symptom ratings, data are missing from up to 15 patients on some ratings, resulting in a smaller size in the statistical analysis on the paired pre- to postoperative symptom ratings. Furthermore, the study would have benefited from a preoperative GERD-HRQL assessment, as the change in the total GERD-HRQL and symptom scores would be of interest. Another limitation is the heterogeneous follow-up period which ranges from 1 to 9 years of follow-up, where the symptom burden and QoL may change significantly over the years.

Data on the use of PPIs were obtained until November 2023, whereas the subjective GERD-HRQL ratings were from 2021. Although the timepoints are different, the data were included as it contributed with more data on the long-term use of PPIs following ARS. The subjective evaluation of the QoL and symptom ratings combined with an objective evaluation in the form of an accurate, register-based estimation of PPI use is a major strength of the study.

A possible reason for the higher GERD-HRQL score, more frequent reflux symptoms and lower satisfaction rates found in this study compared to the literature might be a greater proportion of patients included experiencing PPI refractory symptoms. In Denmark, the rate of use of ARS has been trending downward and possibly toward a more restrictive approach to ARS resulting in surgery being performed primarily in patients that experience GERD without sufficient effect of pharmacological therapy [32]. In the present study, this is supported by the finding of more than 180 DDD of PPIs used by 84% of patients in the year prior to surgery as well as a preoperative median overall severity grading of their symptoms of 8 on a scale of 1–10. In the LOTUS RCT, patients were excluded if they only had a partial or no response to PPI therapy in a 3-month period prior to surgery [25]. An RCT comparing surgical treatment to medical treatment of highly selected PPI refractory patients found a treatment success rate of 67% in the surgical group, superior to the medically treated patients, but a substantially lower success rate than found in typical, PPI-responsive GERD [33]. However, they did find that truly PPI refractory heartburn was only present in a minority of the patients referred as a substantial amount had PPI-responsive GERD when PPIs were taken correctly or had a normal pH monitoring. In the present study, pH monitoring was abnormal in almost all patients and alleviation therapy was sought optimized prior to surgery. Postoperative objective assessments by pH monitoring and HR manometry would be of interest in establishing the cause of the frequent symptoms and high use of PPIs but were not routinely performed postoperatively. These assessments were only performed if indicated by the patients’ symptoms and they were considered for reoperation as part of evaluation and management of failed antireflux surgery [34].

In conclusion, this study found a significant improvement in severity of symptoms of heartburn and regurgitation after a median of 4.8 years of follow-up after ARS, although 32% of patients were experiencing symptoms of heartburn and 29% of regurgitation daily. The symptom severity ratings and their effect on the patients’ perceived QoL were found to be higher compared to the literature. Almost half of patients were not taking any PPIs at follow-up, but 44% became long-term users, and patients should therefore be made aware that continuous long-term PPI can still be the outcome following ARS.
